# Methicillin-Resistant *Staphylococcus aureus* Strains Isolated from Burned Patients in a Tunisian Hospital: Molecular Typing, Virulence Genes, and Antimicrobial Resistance

**DOI:** 10.3390/antibiotics12061030

**Published:** 2023-06-08

**Authors:** Souhir Kmiha, Ahlem Jouini, Nahawend Zerriaa, Safa Hamrouni, Lamia Thabet, Abderrazak Maaroufi

**Affiliations:** 1 Laboratory of Epidemiology and Veterinary Microbiology, Group of Bacteriology and Biotechnology Development, Pasteur Institute of Tunis, University of Tunis El Manar, Tunis 2092, Tunisia; souhirkmiha@yahoo.fr (S.K.);; 2 Laboratory of Microbiology, Center for Traumatology and Major Burns, Rue du 1er Mai, Ben Arous 2013, Tunisia; thabetlamia@gmail.com

**Keywords:** Methicillin-resistant *S. aureus* (MRSA), *mecA* gene, virulence factors, *spa* typing, *agr* typing, Panton-Valentine leukocidin, enterotoxins, Tunisia

## Abstract

Methicillin-resistant *Staphylococcus aureus* (MRSA) is one of the major causes of a variety of infections in hospitals and the community. Their spread poses a serious public health problem worldwide. Nevertheless, in Tunisia and other African countries, very little molecular typing data on MRSA strains is currently available. In our study, a total of 64 MRSA isolates were isolated from clinical samples collected from burned patients hospitalized in the Traumatology and Burns Center of Ben Arous in Tunisia. The identification of the collection was based on conventional methods (phenotypic and molecular characterization). The characterization of the genetic support for methicillin resistance was performed by amplification of the *mecA* gene by polymerase chain reaction (PCR), which revealed that 78.12% of *S. aureus* harbors the gene. The resistance of all the collection to different antibiotic families was studied. Indeed, the analysis of strain antibiotic susceptibility confirmed their multi-resistant phenotype, with high resistance to ciprofloxacin, gentamicin, penicillin, erythromycin, and tetracycline. The resistance to the last three antibiotics was conferred by the *blaZ* gene (73.43%), the *erm*(*C*) gene (1.56%), the *msr*(*A*) gene (6.25%), and *tet*(*M*) gene (7.81%), respectively. The clonal diversity of these strains was studied by molecular typing of the accessory gene regulator (*agr*) system, characterization of the SCC*mec* type, and *spa*-typing. The results revealed the prevalence of *agr* types II and III groups, the SCC*mec* type III and II cassettes, and the dominance of *spa* type t233. The characterization of the eight enterotoxins genes, the Panton-Valentine leukocidin and the toxic shock syndrome toxin, was determined by PCR. The percentage of virulence genes detected was for enterotoxins (55%), *tst* (71.88%), leukocidin E/D (79.69%), and *pvl* (1.56%) factors. Furthermore, our results revealed that the majority of the strains harbor IEC complex genes (94%) with different types. Our findings highlighted the emergence of MRSA strains with a wide variety of toxins, leukocidin associated with resistance genes, and specific genetic determinants, which could constitute a risk of their spread in hospitals and the environment and complicate infection treatment.

## 1. Introduction

The high frequency of *Staphylococcus aureus* infections in burn units constitutes a serious problem for infection treatment. Loss of the functional skin barrier and the depression of the immune responses caused by burns have increased the incidence of various infections [1]. The skin is the first barrier of defense against microbial infection, and it becomes more sensitive once it gets burned. Many pathogens are responsible for burn wound infections, including *Staphylococcus*, *Enterococcus*, *Pseudomonas*, *Acinetobacter*, and fungi [1]. *Staphylococcus aureus* bacteria can cause a wide variety of infections, from minor skin infections to life-threatening infections such as pneumonia, endocarditis, and sepsis. During the last five decades, the overuse of antimicrobial agents in human medicine to treat bacterial infections has favored the emergence of multidrug-resistant bacteria, including MRSA, that have spread as human hospital-acquired pathogens (HA-MRSA) throughout the world [2].

The emergence and transmission of methicillin-resistant *S. aureus* (MRSA) in burn centers results in adverse effects such as prolonged hospitalization, bacteremia or sepsis, and even death, which require further prevention and treatment efforts. In Tunisia, the Traumatology and Burn Center (CTGB) is the only burn center that treats different types of burn wounds. 

Methicillin resistance in staphylococci is primary mediated by the expression of the *mecA* gene, or its homologue *mecC*, that contains a diverse type of staphylococcal cassette chromosome *mec* (SCC*mec*), based on a mobile genetic element, and encodes an altered penicillin-binding protein with a very low affinity to β-lactam antibiotics [2]. This emergence of multidrug-resistant strains presents a global health issue. In fact, the World Health Organization predicts that by 2050, bacterial resistance will be responsible for 10 million more deaths than cancer [3].

To date, there is no staphylococcal vaccine, and the alternative antibiotic used as an anti-MRSA is vancomycin. The main objective for successful clinical treatment of bacterial infections depends on the analysis of the antibiotic susceptibility profiles and the antibiotic resistance mechanisms of pathogenic bacteria. In addition to antibiotic resistance, *S. aureus* isolates can harbor a diversity of virulence factor genes, including the Panton-Valentine leukocidin (PVL), the toxic shock syndrome toxin 1 (TSST), and the staphylococcal enterotoxins (SE) [4], immune evasion factors like staphylokinase (Sak), staphylococcal inhibitor of complement (SCIN), and chemotaxis inhibitory protein (CIP), including CHIPS. These virulence factors may contribute to human or animal skin and soft tissue infections, as well as cases of severe pneumonia and food poisoning [4]. The number and combinations of toxin genes may contribute to the pathogenicity of *S. aureus*. It is important to highlight that the expression of virulence genes is under the control of a global quorum-sensing regulator system named *agr* (accessory gene regulator), which is associated with the pathogenesis and molecular typing of antibiotic resistance in *S. aureus* [5]. Mobile genetic elements (MGEs) carrying resistance genes such as plasmids, transposons, and genomic islands frequently harbor virulence factor genes [2,6]. 

The molecular characterization of *S. aureus* is very interesting for the identification and knowledge of the circulation of virulent and resistant clones in hospital settings [6]. In fact, the most useful typing tool for epidemiological studies of *S. aureus* that gives an excellent discriminatory result is *spa*-typing based on the sequence variation in a hypervariable region of the staphylococcal protein A *spa* gene. The *spa* gene encodes a surface protein that plays a role in the adhesion and colonization of *S. aureus* [7]. The prevalence of *spa* types of *S. aureus* isolates varies among different origins and countries [6,7].

To develop effective control and treatment of human infections, it is important to study the genetic diversity, antimicrobial resistance, and virulence of *S. aureus* associated with infectious diseases. These types of data are limited in Tunisia. Therefore, the aim of this study was to determine the genetic lineages, antibiotic resistance genes, and virulence determinants of *S. aureus* isolates from clinical samples of burned patients in Tunisia.

## 2. Results 

### 2.1. Confirmation of S. aureus Isolates

The biochemical and molecular identification was performed on the sixty-four isolates collected from the Microbiology laboratory of CTGB. All the isolates were identified as *S. aureus* since they presented the ability to coagulate rabbit plasma and were confirmed by a species-specific *nuc* gene PCR assay.

### 2.2. Antibiotic Resistance Rates

**Figure 1:**
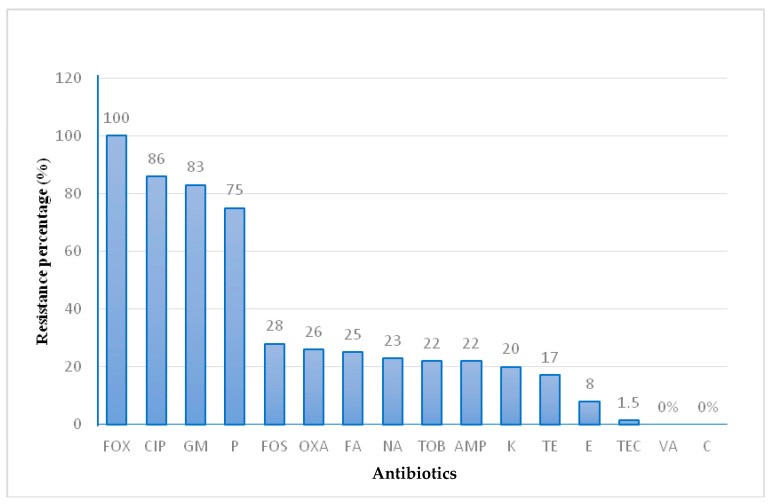
Phenotypic resistance rate of MRSA isolates. Penicillin (P), Oxacillin (OXA), Cefoxitin (FOX), Gentamicin (GM), Kanamycin (K), Tobramycin (TOB), Tetracycline (TE), Ciprofloxacin (CIP), Erythromycin (E), Fusidic Acid (FA), Ampicillin (AMP), Teicoplanin (TEC), Amikacin (NA), Fosfomycin (FOS), Vancomycin (VA) and Chloramphenicol (C).

The occurrence of antibiotic resistance in the 64 *S. aureus* isolates is presented in Figure 1. All *S. aureus* strains were confirmed to be MRSA and presented oxacillin and/or cefoxitin resistance. A high resistance rate for the β-lactam, quinolone, and aminoglycoside families was observed. The percentages were as follows: cefoxitin (100%), ciprofloxacin (86%), gentamicin (83%), and penicillin (75%), while moderate and low resistance rates were detected for: fosfomycin (28%), oxacillin (26%), fusidic acid (25%), amikacin (23%), tobramycin (22%), ampicillin (22%), kanamycin (20%), tetracycline (17%), erythromycin (8%), and teicoplanin (1.5%). Sixty *S. aureus* isolates were multiresistant to at least three antibiotic families.

### 2.3. Genetic Support of Antibiotic Resistance

**Figure 2:**
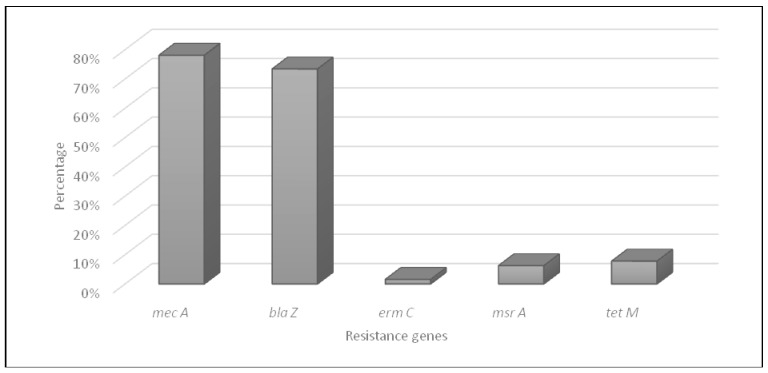
Distribution of antibiotic resistance genes in MRSA isolates.

The characteristics of resistance genes in *S. aureus* isolates are shown in Figure 2. The molecular characterization of methicillin resistance by PCR amplification showed that 50 MRSA strains harbored the *mecA* gene. Penicillin resistance is coded by the *blaZ* gene in 47 MRSA isolates. Furthermore, tetracycline resistance was conferred by *tet*(*M*) genes in only five isolates; *tet*(*L*) and *tet*(*K*) genes were not detected. For the five erythromycin-resistant MRSA isolates, *erm*(*C*) gene was detected in only one strain and *msr*(*A*) gene in four isolates. The *erm*(*A*), *erm*(*B*), and *msr*(*B*) genes were not detected in all the collection.

### 2.4. Molecular Typing of MRSA Isolates

Table 1 presents molecular typing by SCC*mec* cassettes, *spa*-typing, and *agr*-typing. The characterization of SCC*mec* cassettes was performed by multiplex PCR amplification of genes encoding *ccr* recombination. 

The results revealed that 37 of the tested strains (57%) exhibited different *ccr* profiles. Indeed, the most dominant recombination was *ccrA3-ccrB*, assigned to SCC*mec* type III in 22 strains, of which 4 were *mecA* gene negative, followed by *ccrA2-ccrB* recombination assigned to SCC*mec* type II in 13 strains, of which only 1 was *mecA* gene negative. In addition, the *ccrA1-ccrB* recombination allocated to SCC*mec* type I was detected in two MRSA strains, one of which was *mecA*-negative (Table 1).

The analysis of the results of *spa* type for all MRSA strains showed the presence of 10 different *spa* types (*spa* type number of isolates): t233 (20), t2524 (6), t067 (3), t1192 (3), t1209 (3), t037 (3), t2453 (1), t2612 (5), t9082 (1), and t808 (1). Nevertheless, the *spa* type in eighteen strains was not typable.

The amplification of the *agr* locus by multiplex PCR showed the dominance of the *agr* II type in 28 strains, followed by the *agr* III type in 23 strains; the remaining isolates were ascribed to the *agr* I type. 

### 2.5. Virulence Genes and IEC Profile of MRSA Isolates 

**Figure 3:**
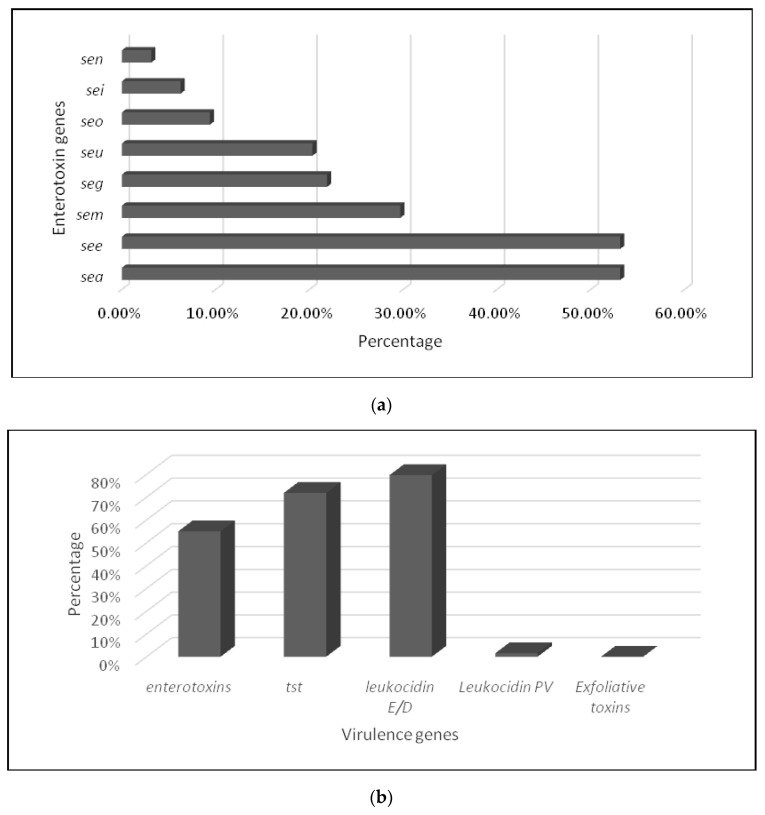
Prevalence of virulence genes in MRSA isolates: (**a**) enterotoxins and (**b**) different virulence genes.

The presence of virulence genes in MRSA isolates is shown in Table 2 and Figure 3.

The multiplex PCR of different virulence factors revealed that 63 MRSA isolates (98.44%) harbored virulence genes. Eight enterotoxin genes were detected (number of isolates, percentage) (Figure 3a): *seg* (14, 21.87%), *sea* (34, 53.12%), *see* (34, 53.12%), *seo* (6, 9.37%), *sem* (19, 29.68%), *sei* (4, 6.25%), *sen* (2, 3.12%), and *seu* (13, 20.31%). While the genes coding for the toxic shock protein TSST were found in 46 (71.88%) strains, four MRSA harbored the gene *lukS-lukF* encoding for PVL and were associated with leukocidin E/D factor in three strains. In addition, 51 isolates carried the gene for leukocidin E/D factor; none of them was *etA*- or *etB*-positive (Figure 3b). Different gene combinations coding the IEC complex were found in almost all *S. aureus* strains (n = 60) and assigned to 3 IEC types: IEC type B with the association of *scn*, *sak*, and *chp*; type E with the association of *scn* and *sak;* and type D with the association of *scn*, *sak*, and *sea*. 

## 3. Discussion

*Staphylococcus aureus* is one of the main causes of nosocomial and hospital infections, leading to serious health problems [2]. Our study focused on the characterization of antibiotic resistance, mainly methicillin resistance, in *S. aureus* strains isolated from different clinical samples of burned patients hosted in the Burn and Traumatology Center in Tunisia. The research on the genetic support of various resistance mechanisms and virulence factors, as well as molecular typing, was intended to provide insight into their clonal diversity and the spread of resistant and virulent clones. Our study included 64 *staphylococcus* strains recovered from clinical samples taken from burned patients. Biochemical and molecular identification revealed that all isolates are *staphylococcus aureus*. The presence of *S. aureus* in burned patients in the Tunisian Center could indicate that this species is involved in cutaneous superinfections of burns due to its commensalism and its diverse virulence factors. Our results are in agreement with those of the study of Thabet et al. [8], performed in two Tunisian hospital structures (CTGB and Aziza Othman Tunis) and reporting that *S. aureus* is the main bacteria isolated in burns in the new hospital center. Its implication in different types of infections was in agreement with recent reports from China, Iran, and Africa describing *S. aureus* as the main pathogen bacteria frequently isolated from burned patients and hospital settings [6].

The analysis of antibiotic resistance in the current study revealed that all strains have been confirmed to be methicillin-resistant. This could be due to the widespread and uncontrolled use of β-lactam drugs, which are the first-line treatment for staphylococcal infection [9]. Our feeding is in agreement with different studies in Tunisia [10,11,12], Morocco [13], and Africa [6]. This resistance to beta-lactams has evolved in successive waves as specific resistance mechanisms have been acquired [14]. In the same context, Fallagas et al. [15] showed that in most of the high and medium Human Development Index countries analyzed, the most pronounced increase was observed in Tunisia, with an increase up to 41–46% after 2005, as compared with a prevalence of 12%–18% years before. Thus far, in South Africa, the prevalence of MRSA decreased from 36% in 2006 to 24% during 2007–2011, probably due to the implementation of effective infection control policies. In Algeria and Egypt, according to the same study, the prevalence of MRSA between 2003 and 2005 was 45% and 52%, respectively. Morocco is the only country where a low prevalence of MRSA seems to have stabilized during 2003–2008.

The MRSA strains showed high resistance to all β-lactam antibiotics. The molecular analysis of methicillin resistance showed that 78% of MRSA isolates harbored the *mecA* gene; the remaining strains (22%) were *mecA*-negative; this suggests that they may contain further variant cassette genes of the *mecA* gene, such as the *mecC*, *mecB*, or *mecD* genes recently detected [2]. Indeed, the dissemination of MRSA encoded by the *mecA* gene in clinical settings has been reported in various African and European countries. This resistance has emerged through the SCC*mec* cassette genes, which can disseminate by horizontal transfer [16].

In addition to the resistance to methicillin, MRSA isolates showed resistance, especially to penicillin, conferred by the *blaZ* gene (73.43% of isolates). The resistance to tetracycline and erythromycin is encoded, respectively, by *tet*(*M*), *erm*(*C*), and *msr*(*A*) genes. Our findings conflict with the study of Zmantar et al. [17], which reported a higher frequency of *erm*(*A*) and *erm*(*C*) genes detected in MRSA isolates from the oral cavity of Tunisian children. Nevertheless, our results were similar to those of Mkhize et al. [17], who described the presence of the *erm*(*C*) gene and the absence of the *erm*(*A*) and *erm*(*B*) genes in *S. aureus* isolates collected from public hospitals in South Africa [18]. 

The characterization of SCC*mec* types in our analyzed collection by multiplex PCR amplification of genes encoding *ccr* recombination revealed that 37 strains (57%) exhibited different *ccr* profiles. Indeed, the most dominant recombination was *ccrA3-ccrB*, assigned to SCC*mec* type III (3A), followed by the *ccrA2-ccrB* recombination assigned to SCC*mec* type II (2A), with only two strains having the *ccrA1-ccrB* recombination (SCC*mec* type I (1A)). Nevertheless, SCC*mec* types I, II, and III were the most common during a study conducted at Charles Nicole Hospital in Tunis [19] and SCC*mec* type IV is the most often reported cassette in the majority of research performed in clinical settings in Tunisia [6,10,11,20].

Furthermore, SCC*mec* type I was also reported in the hospital environment in Tunisia [21]. Chen K. et al. [1] demonstrated that the most prevalent clone of MRSA in the burn center in Southeastern China was ST239-SCC*mec*III-t030. According to research performed in Brazil in 2013, SCC*mec* type III had the highest prevalence in burn units [22]. Nevertheless, another study in Iran reported several types of SCC*mec* (47.5% type III, 25% type IV, 10% type V, 10% type II, and 7.5% type I) in MRSA isolates from burned patients at Motahari Hospital (Iran) [23].

In the present study, the molecular characterization of the polymorphic X region of the *spa* gene showed the presence of 10 different *spa* types (t233, t2524, t067, t1192, t1209, t037, t2453, t2612, t9082, and t808) among 46 MRSA isolates. Noteworthy, the *spa* type t233 was the most prevalent in 20 MRSA isolates. These findings are not consistent with those reported in a recent study that described the distribution of the most prevalent *spa* types in the world. Indeed, in Africa, t037, t064, and t084 are frequently found. In Europe, it is rather the t032, t008, and t002, and in Asia, the *spa* type t037 and t002 are the most often reported [24]. This suggests that the *spa* type t233 could be a new clone circulating in the CTGB hospital environment. 

The molecular typing by amplification of the *agr* locus revealed the dominance of *agr* types II and III, which is in agreement with the study of Kechrid et al. (2011) at the Children’s Hospital of Tunis [11]. On the other hand, the study reported by Elhani et al. [25] as well as that of Gharsa et al. [21] showed that the *agr* I group was the most present. The pathogenic and biofilm growth of *S. aureus* are primarily regulated by the accessory gene regulator (*agr*) quorum-sensing system. In a cell density-dependent manner, this system inhibits the transcription of numerous cell wall-associated proteins, such as protein A and microbial surface components recognizing adhesive matrix molecules (MSCRAMMs), and activates a number of exoproteins, such as hemolysins, exfoliative toxins (ETs), and toxic shock syndrome toxins (TSSTs) [26]. Numerous investigations have documented the association between particular *agr* classes, particular clonal complexes, disease kinds, and associated virulence factors. For instance, Holtfreter et al. [27] discovered a correlation between particular *S. aureus* lineages and different *agr* types. Many studies showed that TSST-1 was predominately carried by *agr* III-type isolates, while phages and plasmids commonly occur in *agr* IV isolates that contain *etA* or *etB* genes [28]. The *agr* I type was also the most typical type, particularly among MRSA isolates [28]. 

Investigation of the virulence determinants and their implication in the infection by MRSA isolates was scarce in Tunisia and Africa. To the best of our knowledge, the current study was the first to characterize the virulence factors in MRSA strains isolated from hospital patients, especially burned ones. Indeed, our findings highlighted the occurrence of MRSA strains with a wider range of virulence genes. The enterotoxin genes were detected in the majority of strains, indicating that despite the fact that enterotoxin genes are encoded by a bacteriophage, the bacteriophage has spread easily among strains with the same genetic background [29]. In addition, we detected that all strains have a high incidence of *sea* and *see* genes, which was in accord with other findings [30].

According to the characterization of virulence factors in our collection, the prevalence of the *lukF/S-PV* gene was low (1.56%). In the same context, Viquez-Molina et al. [31] detected the *lukF/S-PV* gene in 6.9% of *S. aureus* isolated from patients with diabetic foot infections; however, another study from Lisbon did not detect the *lukF/S-PV* gene from the same origin [32]. Many researchers have suggested that the *PVL* locus is carried on a bacteriophage, and this locus is associated with skin infections and occasionally severe necrotizing pneumonia [33,34]. The presence of *PVL* gene in MRSA strains was mostly associated with skin and soft tissue infections and community-associated clones [6]. Reported data from Tunisia and Algeria described high PVL prevalence in MRSA isolates, while studies from South Africa revealed low prevalence [6].

The toxic shock syndrome (TSST), which is generated by *Staphylococcus aureus*, has been associated with a number of acute illnesses [35]. The *tst* gene was found to have a very high percentage in all strains (71.88%). Our results contradicted other research [36] that described a high prevalence of *tst*-carrying isolates among methicillin-susceptible isolates as compared to MRSA isolates, indicating a possible link between the drug resistance of the strains and the occurrence of their virulence genes. In addition, our results reported that *tsst* toxin is produced by MRSA strains affiliated with *agr* types I, II, and III, whereas other research reported that *tsst* is produced preferentially by isolates harboring *agr* III in MRSA strains isolated from hospitals [28].

The genes encoding *etA* and *etB* exfoliatins were no longer detected in our collection; this can be suggested by the fact that the plasmids and phages carrying these exfoliatins are linked to isolates expressing *agr* type IV [28].

There is evidence that some *agr* types are associated with many clinical characteristics. Most toxic shock syndrome (TSST-1) strains, for instance, are classified as *agr* group III, while most strains with leukocidin-induced necrotizing pneumonia are classified as *agr* group II [37,38]. In our study, *lukE/D* was related to *agr* type III. Our findings disagree with the report of Ben Nejma et al. [10], who have revealed that PVL negative strains are classified as *agr* type III. Similarly, Xu et al. [31] demonstrated that all *lukS/F-PV*-positive isolates belong to *agr* group I.

## 4. Material and Methods

### 4.1. Bacterial Isolates

The study was carried out on a collection of 64 non-duplicated *S. aureus* isolates collected from 64 different clinical samples from burned patients hospitalized in the Traumatology and Burn Center (CTGB) of Tunisia between January and December 2016. The clinical samples were distributed as follows: blood culture (18), cytobacteriological examination of sputum (3), puncture (4), nasal pus (1), catheters (6), and sebum (32).

The isolates were recovered on petri dishes of Brain Heart Infusion Agar (BHI) from the Microbiology laboratory of CTGB and transferred to the laboratory in a cooler for analysis. 

### 4.2. Strain Identification

The isolates were identified by conventional biochemical tests (Gram staining oxidase, catalase, DNase, and ability to coagulate rabbit plasma (Bio-Rad, France) [4,39]. Molecular identification was performed by species-specific PCR amplification of the *nuc* gene, as previously described [39], with *S. aureus* (ATCC 43300) being used as a control strain.

### 4.3. Antimicrobial Susceptibility Testing

The determination of antibiotic susceptibility was performed using the disk diffusion method in accordance with the Clinical and Laboratory Standards Institute (CLSI) recommendations [40]. The antimicrobial agents tested (charge in µg) were as follows: penicillin (10), oxacillin (1), cefoxitin (30), vancomycin (30), gentamicin (10), kanamycin (30), tobramycin (10), tetracycline (30), ciprofloxacin (5), erythromycin (15), amikacin, fusidic acid (10), teicoplanin (30), fosfomycin (200), chloramphenicol (30), and ampicillin (30). 

### 4.4. Detection of the mecA Gene 

Methicillin resistance was detected by oxacillin and cefoxitin susceptibilities on disk diffusion agar, according to CLSI [40]. Confirmation of methicillin resistance was performed by conventional PCR targeting the *mecA* gene [41]. *S. aureus* ATCC 43300 was used as a control strain. 

### 4.5. SCCmec-Typing in MRSA Isolates

The presence of SCC*mec* types I to V was investigated in MRSA isolates by PCR of the *ccr* recombinases (1–5) and the *mec* gene complex type (A to C), as recommended by the International Working Group on the Classification of Staphylococcal Cassette Chromosome Elements (IWG-SCC) [42] (Table S1).

### 4.6. Molecular Typing of S. aureus Isolates

*S. aureus* protein A *spa*-typing was performed in all *S. aureus* isolates (n = 64) as elsewhere described [43]. The polymorphic X region of the *spa* gene was amplified by PCR, sequenced, and analyzed using Ridom *staph*-type software version 1.5.21 (Ridom GmbH). It automatically detects *spa* repeats and assigns a *spa* type, according to http://spaserver.ridom.de/ (accessed on 10 December 2005). To determine the type of *agr* in MRSA, two multiplex PCRs were performed; the first one allowed the amplification of *agr* types I and II, and the second PCR amplified *agr* types III and IV [44].

### 4.7. Detection of Antimicrobial Resistance Genes

The detection of antimicrobial resistance genes (*blaZ*, *erm*(*A*), *erm*(*B*), *erm*(*C*), *msr*(*A*), *msr*(*B*), *tet*(*K*), *tet*(*M*), *and tet*(*L*)) was investigated in resistant isolates by specific PCRs [45]. Positive and negative controls used in each PCR assay were from the collection of the laboratory of Institute Pasteur of Tunis.

### 4.8. Detection of Staphylococcal Toxin Genes

All isolates were tested by PCR for the presence of genes coding for the various staphylococcal enterotoxins (*sea*, *see*, *seg*, *sei*, *sem*, *sen*, *seo*, and *seu*), toxic shock syndrome toxin 1 (*tst*), leukocidin of Panton Valentine (PVL, *lukF-lukS-PV*), and exfoliative ETA and ETB toxins (*etA* and *etB*) [46]. 

### 4.9. Detection of the Immune Evasion Gene Cluster

All isolates were tested by PCR for the presence of five genes (*scn*, *chp*, *sak*, *sea*, and *sep*) of the immune evasion cluster (IEC) system. These genes are enclosed in the φ3 bacteriophage and encode the IEC system, which helps bacteria survive in the human host by evading the innate immune system. Detected alleles allowed the classification of seven IEC types (from A to G) [47], Table S2.

## 5. Conclusions 

In this study, all the isolates from clinical samples of burned patients were confirmed as MRSA with high rates of resistance to ciprofloxacin and gentamicin conferred by different antibiotic resistance genes. In addition, our data reported the detection of resistance genes and a different virulence profile in MRSA isolates. It is important to report the molecular diversity of *spa* and *agr* types in the study collection. 

This genetic diversity can lead to the emergence and spread of virulent and drug resistant clones within the hospital. Therefore, enhanced antimicrobial surveillance efforts are needed to control and regulate the use of antimicrobials in Tunisian hospital settings in order to reduce the risks associated with the acquisition of multidrug-resistant clones containing virulence determinants and the spread of pathogenic bacteria in humans and their environment. 

## Figures and Tables

**Table 1:** Molecular typing of MRSA isolates by *ccr* genes, *spa* type, and *agr* type.

Reference of Strains	Origin	System *agr*	*ccr*	*spa* Type	SCC*mec*	*mec* Complex
D354	SV	III	*ccrA2-ccrB*	t233	II	A
H2071/2073	Bc	II	─	NT	─	─
D544	PC	III	─	t067	─	─
D905	PC	III	*ccrA3-ccrB*	t1209	III	A
D1069	PC	III	*ccrA3-ccrB*	NT	III	A
D2435	PB	III	*ccrA1-ccrB*	t067	I	B
D21	PB	III	*ccrA2-ccrB*	NT	II	A
H1240	Bc	II	*ccrA3-ccrB*	NT	III	A
D210	PB	I	*ccrA3-ccrB*	t2453	III	A
D1462	PB	II	*ccrA2-ccrB*	t9082	II	A
H1066	Bc	III	─	NT	─	─
D1719	PC	III	*ccrA2-ccrB*	t233	II	A
D1434	PC	III	─	t1209	─	─
D1065	PC	II	*ccrA3-ccrB*	t067	III	A
D976	PC	I	─	t2524	─	─
D847	PC	II	*ccrA2-ccrB*	NT	II	A
D2367	PC	II	─	t233	─	─
1039	PC	II	*ccrA2-ccrB*	t1209	II	A
H3720/3772	Bc	II	*ccrA2-ccrB*	t233	II	A
D2085	PC	II	*ccrA3-ccrB*	t233	III	A
H930	Bc	I	*ccrA2-ccrB*	NT	II	A
D1467	PC	II		t2612	─	─
D2376	PC	II	*ccrA2-ccrB*	NT	II	A
H405	Bc	I	─	NT	─	─
H745	Bc	I	*ccrA3-ccrB*	NT	III	A
D2377	PC	II	*ccrA2-ccrB*	t037	II	A
H3715	Bc	III	*ccrA3-ccrB*	t2612	III	A
D2504	KTY	III	*ccrA3-ccrB*	t2612	III	A
D1060	PC	III	*ccrA3-ccrB*	NT	III	A
H950	Bc	III	*ccrA2-ccrB*	t233	II	A
D1114	PC	II	─	t233	─	─
D1829	SV	III	*ccrA3-ccrB*	t233	III	A
D60	KTY	III	─	t233	─	─
D942	CBES	I	─	t233	─	─
D675	PC	III	─	t233	─	─
D1880	PC	II	─	t233	─	─
D1388	PC	III	─	t233	─	─
H794	Bc	II	─	t037	─	─
D1095	NP	II	─	t233	─	─
D1124	PC	III	─	t2524	─	─
H73	Bc	II	─	t233	─	─
H3741	Bc	II	*ccrA3-ccrB*	t2612	III	A
D2240	PC	II		t2612	─	─
D2252	PC	II	*ccrA2-ccrB*	t2524	II	A
H814	Bc	III	*ccrA1-ccrB*	NT	I	B
D1691	PC	I	─	t233	─	─
D1363	KTY	III	*ccrA3-ccrB*	t808	III	A
D491	CBES	I	*ccrA3-ccrB*	NT	III	A
H2268	PC	I	─	t037	─	─
H2879	Bc	II	*ccrA3-ccrB*	NT	III	A
D2187	PC	III	*ccrA3-ccrB*	t2524	III	A
D48	KTY	III	*ccrA3-ccrB*	t233	III	A
D1128	PC	I	*ccrA3-ccrB*	NT	III	A
D1971	KTY	I	*ccrA3-ccrB*	NT	III	A
D890	KTY	I	─	NT	─	─
D1747	PC	II	─	t1192	─	─
H3008	Bc	II	*ccrA3-ccrB*	t1192	III	A
D1077	PC	II	─	t2524	─	─
H1042	Bc	III	*ccrA2-ccrB*	t1192	II	A
D2393	PC	I	─	t2524	─	─
H793	Bc	II	*ccrA3-ccrB*	t233	III	A
H782	Bc	II	─	NT	─	─
D1033	CBES	II	─	t233	─	─
D1836/1795	PC	II	─	t233	─	─

Bc: Blood culture; PC: Post cibum; PB: Puncture; CBES: Cytobacteriological examination of sputum; NP: Nasal pus; KTY, KTV: Catheter; SV: Septum. NT: not typable; SCC*mec*: Staphylococcal Cassette Chromosome *mec*; *Spa*: *S. aureus* protein A; *agr*: accessory gene regulator; *ccr*: cassette chromosome recombinase.

**Table 2:** Resistance phenotype profiles, resistance genes, *agr*-typing, virulence factors, and the Immune Evasion Cluster detected in MRSA isolates.

Reference of Strains	Origin	Antibiotic-Resistant Phenotype	Resistance Genes Detected	Virulence Factors	*agr* System
D354	SV	OXA, CIP, GM, FOX, FA	*mecA-blaZ*	*tst*, *seg*, *sea*, *see*, IEC B	III
H2071/2073	Bc	OXA, CIP, GM, FOX, FA	*mecA-blaZ*	IEC E, *seo*, *tst*	II
D544	PC	FOX, OXA	*mecA*	leucocidin E/D, IEC B, *sem*, *sea*, *see*, *tst*	III
D905	PC	OXA, CIP, GM, FOX	*mecA-blaZ*	*sei*, *sea*, *see*, *seo*, *tst*, IEC B	III
D1069	PC	OXA, FA, CIP, GM, FOX	*blaZ*	*tst*, IEC B	III
D2435	PB	OXA, FOX	*blaZ*	*tst*	III
D21	PB	OXA, FOX, FA	*mecA*	leucocidin E/D, Panton Valentine leucocidin, *tst*, IEC D	III
H1240	Bc	OXA, FA, GM, FOX	*mecA-blaZ*	*tst*, IEC E	II
D210	PB	OXA, FA	*mecA*	*tst*, IEC E	I
D1462	PB	OXA, P, CIP, GM, TOB, K, FOX, NA, AMP, TE, E	*mecA-blaZ-erm(C)*	leucocidin E/D, Panton Valentine leucocidin, *tst*, *sem*, *sea*, *see*, IEC B	II
H1066	Bc	OXA, FA, CIP, FOX	*mecA-blaZ*	leucocidin E/D, *tst*, IEC D	III
D1719	PC	CIP, GM, FOX	*mecA-blaZ*	leucocidin E/D, *tst*, IEC (*scn*, *sak*, *sea*, *sep*)	III
D1434	PC	OXA, CIP, GM, FOX	*blaZ*	leucocidin E/D, *tst*, IEC B	III
D1065	PC	OXA, FOX	*mecA-blaZ*	leucocidin E/D, *tst*, IEC (*sak*, *chp*)	II
D976	PC	FA, CIP, GM, FOX	*mecA-blaZ*	leucocidin PV, *tst*, IEC E	I
D847	PC	OXA, P, CIP, GM, TOB, K, FOX, NA, AMP, TE	*mecA-blaZ*	leucocidin E/D, *tst*, *sem*, *sea*, *see*, IEC B	II
D2367	PC	CIP, GM, FOX	*mecA*	leucocidin E/D, *tst*, *sem*, *sea*, *see*, IEC B	II
1039	PC	OXA, CIP, GM, FOX	*mecA-blaZ*	leucocidin E/D, *tst*, *sem*, *sea*, *see*, IEC B	II
H3720/3772	Bc	CIP, GM, FOX	*mecA-blaZ*	leucocidin E/D, IEC E	II
D2085	PC	CIP, GM, FOX	*mecA-blaZ*	leucocidin E/D, *tst*, *sem*, *sea*, *see*, IEC B	II
H930	Bc	GM, FOX, TEC	*mecA-blaZ*	leucocidin E/D, *tst*, IEC E	I
D1467	PC	FOX, CIP, GM, FOX	*mecA-blaZ*	leucocidin E/D, *tst*, IEC B	II
D2376	PC	FOX, NA, AMP, P	*blaZ*	leucocidin E/D, *tst*, *sei*, *seo*, *sea*, *see*, IEC B	II
H405	Bc	FOS, FA, CIP, FOX	*mecA-blaZ*	leucocidin E/D, *tst*, IEC B	I
H745	Bc	FOS, FA, CIP, FOX, GM	*mecA-blaZ*	leucocidin E/D, *tst*, *sen*, *sea*, *see*, IEC (*chp*)	I
D2377	PC	FOS, CIP, FOX	*mecA-blaZ*	leucocidin E/D, *tst*, *seo*, *sea*, *see*, IEC B	II
H3715	Bc	FOS, FA, CIP, FOX, GM	*mecA-blaZ*	leucocidin E/D, *tst*, *seg*, *sem*, *see*, *sea*, IEC B	III
D2504	KTY	FOS, FA, CIP, FOX, GM	*mecA-blaZ*	leucocidin E/D, *tst*, *seg*, *sem*, *see*, *sea*, IEC B	III
D1060	PC	CIP, GM, TOB, K, FOX, NA,	*mecA-blaZ*	leucocidin E/D, *tst*, *seg*, *sem*, *see*, *sea*, IEC B	III
H950	Bc	CIP, GM, FOX	*mecA-blaZ*	leucocidin E/D, *tst*, IEC B	III
D1114	PC	CIP, GM, FOX	-	leucocidin E/D, *tst*, *seg*, *sem*, *see*, *sea*, IEC E	II
D1829	SV	CIP, GM, FOX	*mecA*	leucocidin E/D, *seg*, *sem*, *see*, *sea*, *sen*, IEC (*sak*, *chp*)	III
D60	KTY	CIP, GM, FOX	*mecA-blaZ*	leucocidin E/D, *seg*,*sem*, *see*, *sea*, IEC (*sak*, *chp*, *scn*, *sea*)	III
D942	CBES	CIP, GM, FOX	*mecA-blaZ*	leucocidin E/D, *seg*, *seu*, *see*, *sea*, IEC B	I
D675	PC	CIP, GM, FOX	*mecA*	leucocidin E/D, *tst*, *sei*, *seo*, IEC (*sak*, *scn*, *sep*, *sea*)	III
D1880	PC	CIP, GM, FOX	*mecA*	leucocidin E/D, *tst*, *seg*, *sem*, *seu*, *sea*, *see*, IEC (*sak*)	II
D1388	PC	CIP, GM, FOX	*blaZ*	leucocidin E/D, Panton Valentine leucocidin, *tst*, *seo*, *sem*, *seu*, *sea*, *see*, *sei*, IEC B	III
H794	Bc	FOS, CIP, FOX	*mecA*	leucocidin E/D, *sem*, *see*, *sea*, IEC (*sak*, *chp*)	II
D1095	NP	FA, CIP, GM, TOB, K, FOX, NA, AMP, P, TE, E	*mecA-msrA*	leucocidin E/D, *sem*, *see*, *sea*, *seu*, IEC (*sak*, *chp*)	II
D1124	PC	FA, CIP, GM, FOX	*mecA*	leucocidin E/D, *tst*, IEC B	III
H73	Bc	CIP, GM, FOX	*mecA*	leucocidin E/D, IEC (*sea*)	II
H3741	Bc	CIP, GM, FOX, FOS	*mecA*	leucocidin E/D, IEC (*sak*, *chp*, *scn*)	II
D2240	PC	CIP, GM, FOX, FOS	*mecA-blaZ*	leucocidin E/D, IEC (*chp*)	II
D2252	PC	CIP, GM, FOX, FA	*mecA*	leucocidin E/D, IEC (*scn*), *tst*	II
H814	Bc	CIP, GM, FOX, FOS, FA	*mecA-blaZ*	leucocidin E/D, IEC (*scn*), *tst*, *sea*, *see*	III
D1691	PC	CIP, GM, FOX	*-*	leucocidin E/D, *tst*, IEC B	I
D1363	KTY	FA, CIP, GM, TOB, K, FOX, AMP, P, TE	*mecA-blaZ*	leucocidin E/D, *tst*, *sem*, *see*, *seu*, *sea*, IEC D	III
D491	CBES	CIP, GM, TOB, K, FOX, NA, AMP, TE	*blaZ*	*tst*, *sem*, *see*, *seu*, *sea*, IEC B	I
H2268	PC	CIP, GM, TOB, K, FOX, NA, AMP, TE, FOS, P, E	*mecA-blaZ-tet(M)-msr(A)*	-	I
H2879	Bc	CIP, GM, TOB, FOX, NA, AMP, TE, FOS, P, E	*mecA-blaZ-msrA*	leucocidin E/D, IEC (*sak*), *tst*	II
D2187	PC	CIP, GM, FOX, FOS	*mecA-blaZ*	leucocidin E/D, *tst*	III
D48	KTY	CIP, GM, FOX, NA	*blaZ*	leucocidin E/D, *tst*	III
D1128	PC	CIP, GM, TOB, K, FOX, NA, AMP, P	*mecA-blaZ*	leucocidin E/D, *tst*, IEC B	I
D1971	KTY	CIP, GM, TOB, K, FOX, NA, AMP, P, TE, E	*mecA*-*blaZ*-*tet(M)-msrA*	leucocidin E/D, *tst*, IEC B	I
D890	KTY	CIP, GM, TOB, K, FOX, NA, AMP, P	*mecA-blaZ*	*tst*, *seg*, *seu*, *sea*, *see*, IEC B	I
D1747	PC	CIP, GM, TOB, K, FOX, NA, AMP, P, TE	*blaZ-tet(M)*	*sem*, *seu*, *sea*, *see*, IEC B	II
H3008	Bc	CIP, GM, FOX, TOB, K, NA, AMP, P, TE	*mecA*-*blaZ*-*tet(M)*	leucocidin E/D, *seg*, *seu*, *sea*, *see*, IEC (*chp*)	II
D1077	PC	GM, FOX, CIP, FOS	*mecA-blaZ*	*tst*, IEC (*sak*, *chp*)	II
H1042	Bc	CIP, GM, TOB, K, FOX, NA, AMP, P, TE	*mecA*-*blaZ*-*tet(M)*	leucocidin E/D, *sem*, *seu*, *sea*, *see*, IEC B	III
D2393	PC	GM, FOX, CIP, FOS	*mecA*	*seg*, *seu*, *sea*, *see*, IEC B	I
H793	Bc	GM, FOX, CIP	*blaZ*	leucocidin E/D, *seg*, *seu*, *sea*, *see*, IEC B	II
H782	Bc	GM, FOX, CIP, FOS, FA	-	leucocidin E/D, *sea*, *see*, IEC (*chp*)	II
D1033	CBES	GM, FOX, CIP	*blaZ*	leucocidin E/D, *seg*, *seu*, *sea*, *see*, IEC B	II
D1836/1795	PC	GM, FOX, CIP	*blaZ*	leucocidin E/D, *seu*, *sea*, *see*, IEC E	II

Bc: Blood culture; PC: Post cibum; PB: Puncture; CBES: Cytobacteriological examination of sputum; NP: Nasal pus; KTY, KTV: Catheter; SV: Septum. Enterotoxin genes (*sem*, *seu*, *sea*, *see*, *seg*), toxic shock syndrome toxin 1 (*tst*), immune evasion cluster (IEC) system, leukocidin of Panton Valentine (PVL, *lukF-lukS*-PV)*. mecA*: gene encoding methicillin resistance; *blaZ*: gene encoding penicillin resistance; *tet*(*M*): gene encoding tetracycline resistance; *msr*(*A*): gene encoding erythromycin and clindamycin resistance; *erm*(*C*): gene encoding erythromycin resistance. Penicillin (P), Oxacillin (OXA), Cefoxitin (FOX), Gentamicin (GM), Kanamycin (K), Tobramycin (TOB), Tetracycline (TE), Ciprofloxacin (CIP), Erythromycin (E), Fusidic Acid (FA), Ampicillin (AMP), Teicoplanin (TEC), Amikacin (AN), Fosfomycin (FOS).

## Data Availability

Not applicable.

## Supplementary Materials

The following supporting information can be downloaded at: https://www.mdpi.com/article/10.3390/antibiotics12061030/s1. Table S1: Different SCCmec types identified in S. aureus. Table S2: IEC type detected according to the combination of five genes (*scn*, *chp*, *sak*, *sea*, and *sep*) of the immune evasion cluster (IEC) system in *S. aureus*.
