# Supplementary material for: Methicillin-Resistant Staphylococcus aureus Strains Isolated from Burned Patients in a Tunisian Hospital: Molecular Typing, Virulence Genes, and Antimicrobial Resistance
Source: Antibiotics (Basel). 2023 Jun 8;12(6):1030. doi: 10.3390/antibiotics12061030 (PMC10294828; doi:10.3390/antibiotics12061030)

Table S1: Different SCC*mec* types identified in *S. aureus* (www.SCC*mec*.org; [42]).

Type SCC*mec*	Type *ccr*	Complexe *mec*
**I**	1 *(ccrA1ccrB1)*	B
**II**	2 *(ccrA2ccrB2)*	A
**III**	3 *(ccrA3ccrB3)*	A
**IV**	2 *(ccrA2ccrB2)*	B
**V**	5 *(ccrC)*	C2
**VI**	4 *(ccrA4ccrB4)*	B
**VII**	5 *(ccrC)*	C1
**VIII**	4 *(ccrA4ccrB4)*	A
**IX**	1 *(ccrA1ccrB1)*	C2
**X**	7 *(ccrA1ccrB6)*	C1
**XI**	8 *(ccrA1ccrB3)*	E

Table S2 : IEC type detected according the combinaison of five genes (*scn, chp, sak, sea and sep*) of the immune evasion cluster (IEC) system in *S. aureus* [47].

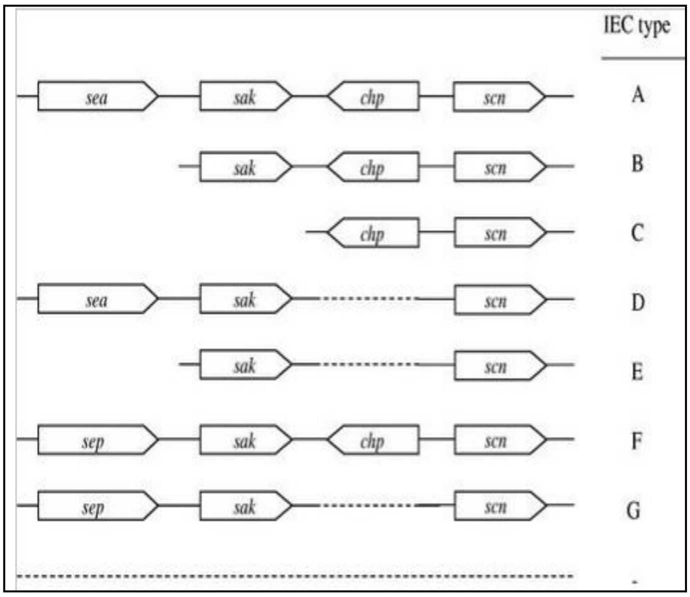

Supplement: Supplementary file 1 [file antibiotics-12-01030-s001.zip › antibiotics-2206135-supplementary-updated.pdf]
